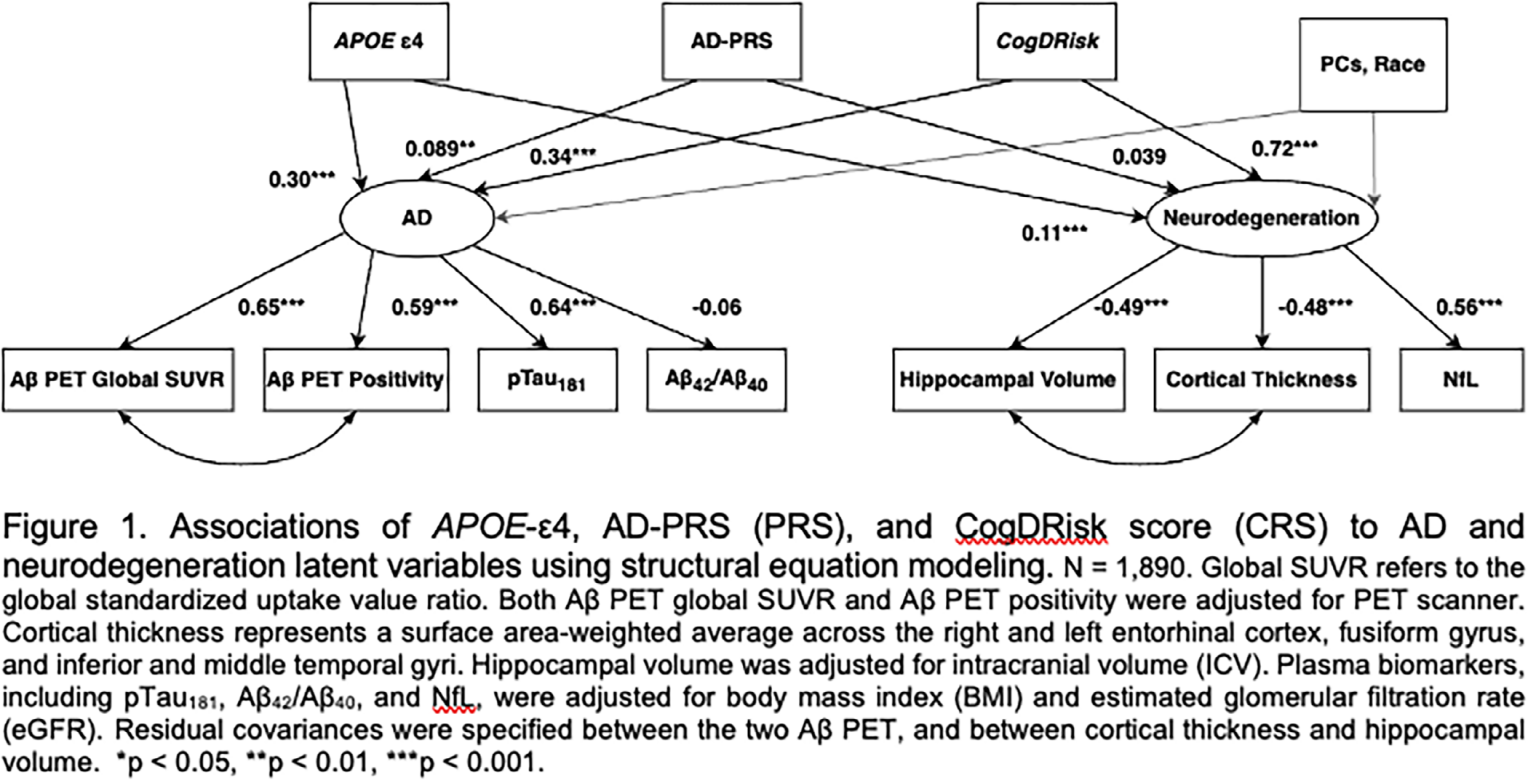# Orthogonal Contributions of Genetic and Clinical Risk Factors on Alzheimer’s Disease and Neurodegenerative Pathogenesis

**DOI:** 10.1002/alz70861_108473

**Published:** 2025-12-23

**Authors:** Meri Okorie, Xiaqing Jiang, Rakshya U Sharma, Ana I Boeriu, Caroline Jonson, Kristine Yaffe, Jennifer S. Yokoyama, Shea J Andrews, Healthy Aging Brain Study ‐ Health Disparities

**Affiliations:** ^1^ University of California, San Francisco, San Francisco, CA USA; ^2^ Department of Psychiatry and Behavioral Sciences, University of California, San Francisco, San Francisco, CA USA; ^3^ Global Brain Health Institute, Memory and Aging Center, University of California San Francisco, San Francisco, CA USA; ^4^ Department of Psychiatry, University of California San Francisco, San Francisco, CA USA; ^5^ University of California, San Francisco, Weill Institute for Neurosciences, San Francisco, CA USA; ^6^ NCIRE‐The Veterans Health Research Institute, San Francisco, CA USA; ^7^ Weill Institute for Neurosciences, University of California San Francisco, San Francisco, CA USA; ^8^ Department of Neurology, University of California, San Francisco, San Francisco, CA USA; ^9^ University of California San Francisco / San Francisco VA Medical Center, San Francisco, CA USA; ^10^ San Francisco Veterans Affairs Health Care System, San Francisco, CA USA; ^11^ Department of Epidemiology, University of California, San Francisco, San Francisco, CA USA; ^12^ Department of Psychiatry, Neurology, and Epidemiology and Biostatistics University of California San Francisco School of Medicine, San Francisco, CA USA; ^13^ University of California, San Francisco and San Francisco VA Health Care System, San Francisco, CA USA; ^14^ Global Brain Health Institute, University of California, San Francisco, San Francisco, CA USA; ^15^ Memory and Aging Center, Weill Institute for Neurosciences, University of California San Francisco, San Francisco, CA USA; ^16^ Global Brain Health Institute (GBHI), University of California San Francisco (UCSF); & Trinity College Dublin, San Francisco, CA USA; ^17^ Department of Radiology and Biomedical Imaging, University of California, San Francisco, San Francisco, CA USA; ^18^ Department of Psychiatry and Behavioral Sciences, University of California ‐ San Francisco, San Francisco, CA USA; ^19^ Icahn School of Medicine at Mount Sinai, New York, NY USA; ^20^ MSSM, New York, CA USA; ^21^ Ronald M. Loeb Center for Alzheimer's Disease, Dept. of Neuroscience, Icahn School of Medicine at Mount Sinai, New York, NY USA; ^22^ Australian National University, Canberra, CA USA

## Abstract

**Background:**

Alzheimer’s disease (AD) is a multifactorial disease influenced by both genetic and clinical risk factors, yet how these factors are associated with AD‐specific or neurodegeneration pathologies remain unclear. Clinical risk scores (CRS) reflect susceptibility to cognitive decline based on lifestyle and other modifiable factors. Genetically, *APOE* ε4 is the strongest known risk factor for AD, but additional genetic contributions can be captured through polygenic risk scores (PRS). This study investigates how *APOE*‐ε4, PRS, and CRS independently contribute to amyloid‐β/tau (i.e. AD) and neurodegenerative pathways in the Healthy Aging Brain Study–Health Disparities (HABS‐HD) cohort.

**Methods:**

In HABS‐HD (*N* =1,890; mean age=67±7.7; African‐descent=328, Amerindian‐descent=647, European‐descent=915), we constructed AD‐PRS using PRS‐CSx‐auto with ancestry‐specific AD genome‐wide association study summary statistics from European, African American, East Asian, and Caribbean Hispanic populations. The Cognitive Health and Dementia Risk Assessment score (CogDrisk), a CRS, was calculated using 17 risk factors for dementia (e.g. age, sex, education, hypertension). Structural equation modeling was used to derive an AD latent variable, indicated by plasma /Aβ_40_ ratio, plasma pTau_181_, Aβ PET positivity, and global SUVR; and a neurodegeneration latent variable, indicated by plasma NfL, cortical thickness, and hippocampal volume (Figure 1). Latent variables were regressed on AD‐PRS, CogDrisk, and *APOE*‐ε4 status, adjusting for race and population stratification.

**Results:**

*APOE*‐ε4 was significantly associated with both AD (β=0.30, *p* <0.001) and neurodegeneration latent variables (β=0.11, *p*<0.001). AD‐PRS was significantly associated with the AD (β=0.089, *p*=8.22e‐3) but not with the neurodegeneration latent variable (β=0.039, *p*=0.21). In contrast, CogDrisk was significantly associated with both the AD (β=0.34, *p*<0.001) and the neurodegeneration latent variables (β=0.72, *p*<0.001).

**Conclusions:**

*APOE*‐ε4 and CRS captures changes related to both neurodegeneration and AD neuropathological proteins, whereas PRS is more specific to AD with the smallest effects. *APOE*‐ε4 remains as the stronger genetic predictor. These findings highlight distinct yet orthogonal roles of genetic and clinical risk factors in dementia pathogenesis.